# New records of Gerromorpha and Nepomorpha (Insecta: Hemiptera: Heteroptera) from South America

**DOI:** 10.3897/BDJ.4.e7975

**Published:** 2016-05-13

**Authors:** Felipe Ferraz Figueiredo Moreira, Higor D. D. Rodrigues, Julianna Freires Barbosa, Barbora Reduciendo Klementová, Marek Svitok

**Affiliations:** ‡ Fundação Oswaldo Cruz, Laboratório de Biodiversidade Entomológica/IOC, Rio de Janeiro, Brazil; § Universidade de São Paulo, Museu de Zoologia, São Paulo, Brazil; | Universidade Federal do Rio de Janeiro, Laboratório de Entomologia, Rio de Janeiro, Brazil; ¶ Department of Biology and General Ecology, Faculty of Ecology and Environmental Sciences, Technical University in Zvolen, Zvolen, Slovakia; # Eawag Swiss Federal Institute of Aquatic Science and Technology, Department of Aquatic Ecology, Centre of Ecology, Evolution and Biogeochemistry, Kastanienbaum, Switzerland

**Keywords:** Aquatic insects, geographic distribution, Neotropical Region, Venezuela, Brazil, Peru.

## Abstract

**Background:**

Aquatic and semiaquatic Heteroptera occur on all continents except Antarctica and occupy a wide variety of habitats, including lentic and lotic water bodies, perennial or temporary. In the Neotropical Region, there is a lack of knowledge concerning the geographical distribution of most represented species, which can only be solved by the collection of specimens in under-studied areas and publication of new records and lists of species.

**New information:**

New records are presented for eleven species of Gerromorpha and ten Nepomorpha, including first records from Venezuela (*Brachymetra lata*, *Limnogonus hyalinus*, *Rhagovelia evidis*, *Tenagobia peruana*, *Limnocoris burmeisteri*, *L. fittkaui fittkaui*, *Placomerus micans*, and *Martarega gonostyla*), the Venezuelan State of Bolívar (*Cylindrostethus palmaris*, *R. elegans*, *R. tenuipes*, and *Ambrysus stali*), the Brazilian State of Bahia (*Martarega bentoi*), Peru (*Euvelia lata*), and the Peruvian Region of Arequipa (*Microvelia pulchella*).

## Introduction

Heteroptera is an interesting group of insects distributed worldwide, being more diverse in tropical zones (Mazzucconi et al. 2009). Most species are terrestrial, but many others are aquatic or semiaquatic, occurring on all continents except Antarctica (Polhemus and Polhemus 2008). Out of the seven infraorders of the suborder, the truly aquatic bugs (most of which live inside the water) are representatives of Nepomorpha, and primarily semiaquatic species constitute Gerromorpha and Leptopodomorpha (Nieser and Melo 1997, Polhemus and Polhemus 2008). The Neotropical fauna of aquatic and semiaquatic Heteroptera is relatively well-known (Polhemus and Polhemus 2007) and there has been increasing interest by research groups from Colombia, Brazil, and Argentina (Moreira 2015). Despite this, several undescribed species occur in the region and there are significant areas which have never been sampled (Polhemus and Polhemus 2007, Polhemus and Polhemus 2008, Moreira 2015). We present new records for 21 species based on material recently collected from understudied areas in Venezuela, Brazil, and Peru.

## Materials and methods

**Figure 1.**
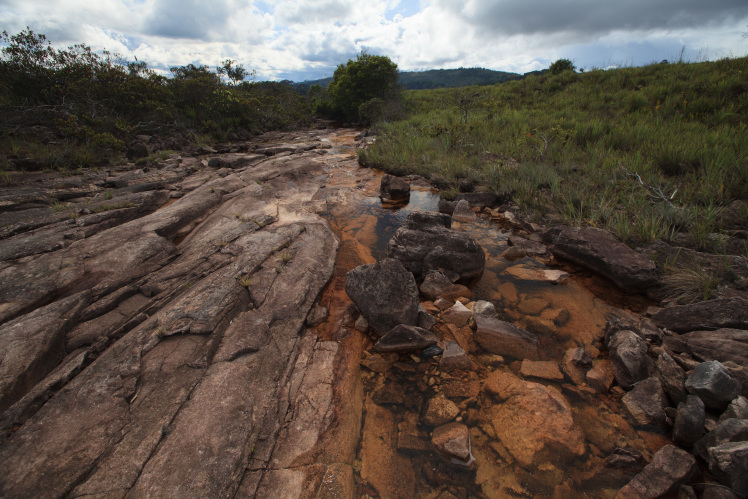
Photograph of collecting locality Biocor 01 in Bolívar State, Venezuela.

**Figure 2.**
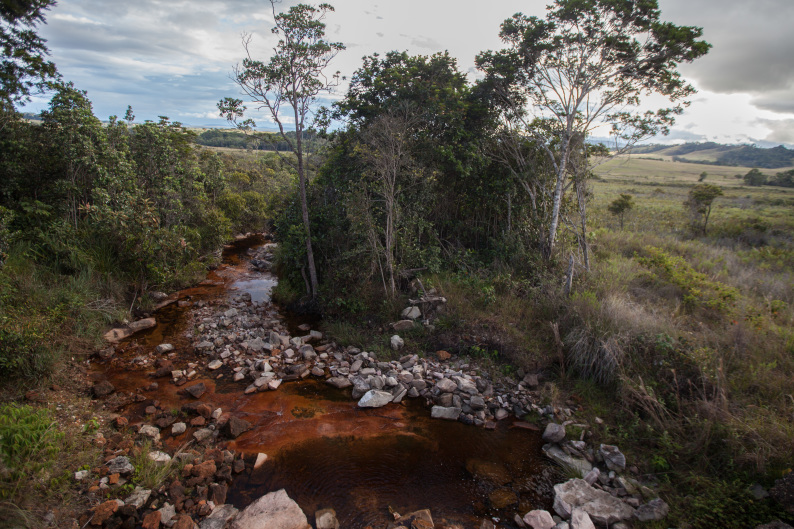
Photograph of collecting locality Biocor 02 in Bolívar State, Venezuela.

**Figure 3.**
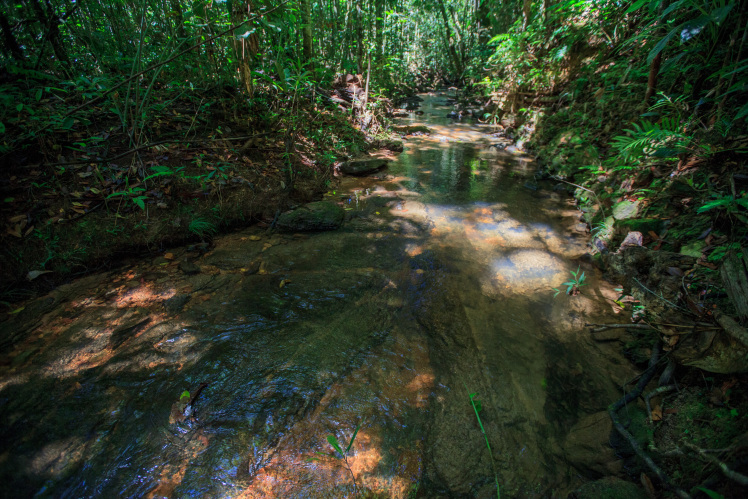
Photograph of collecting locality Biocor 03 in Bolívar State, Venezuela.

**Figure 4.**
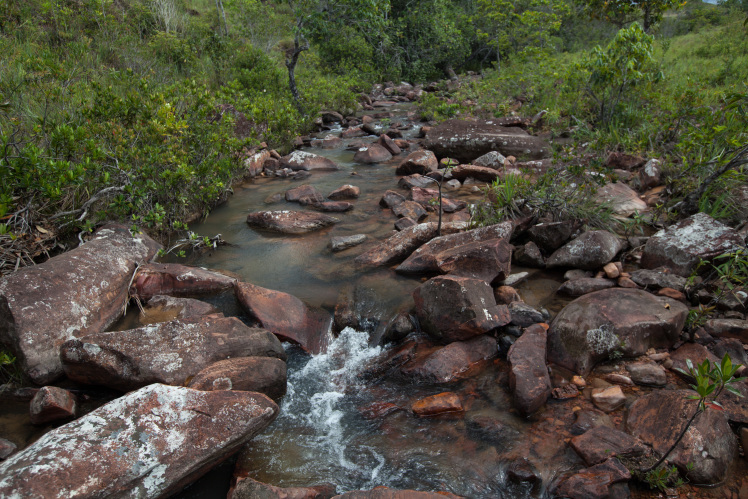
Photograph of collecting locality Biocor 04 in Bolívar State, Venezuela.

**Figure 5.**
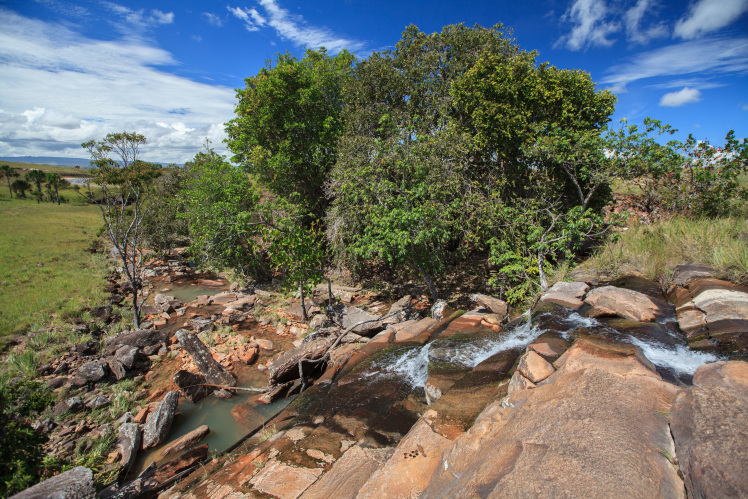
Photograph of collecting locality Biocor 06 in Bolívar State, Venezuela.

**Figure 6.**
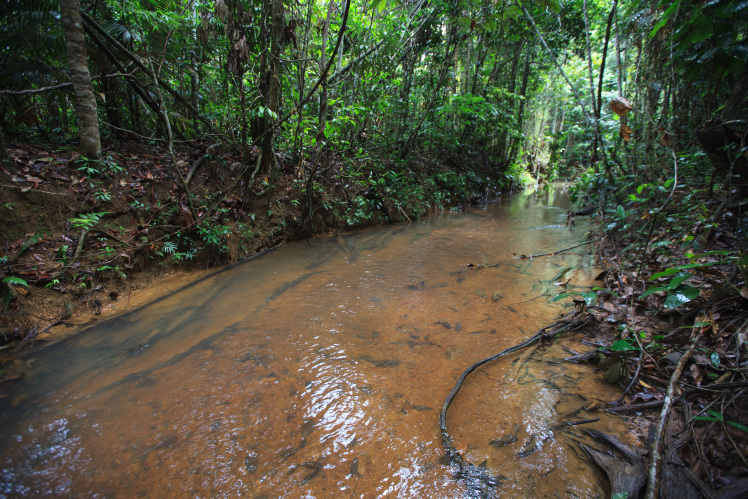
Photograph of collecting locality Biocor 08 in Bolívar State, Venezuela.

**Figure 7.**
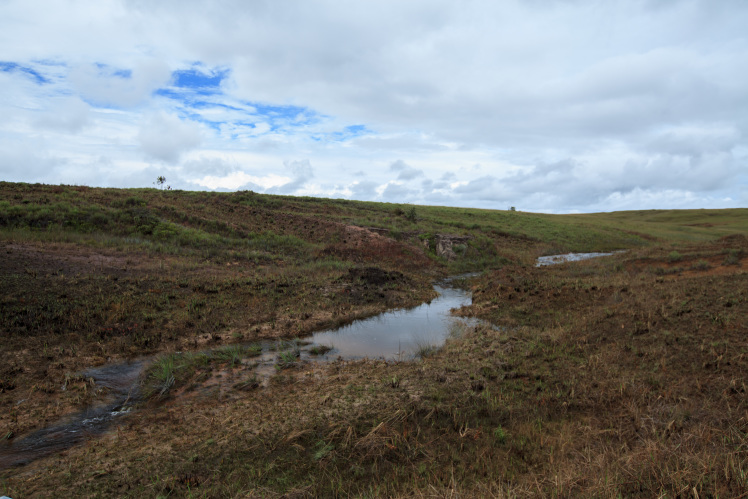
Photograph of collecting locality Biocor 10 in Bolívar State, Venezuela.

**Figure 8.**
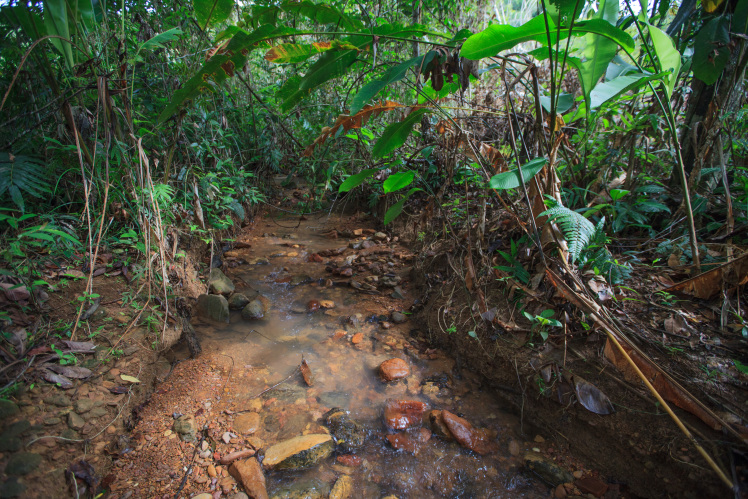
Photograph of collecting locality Biocor 11 in Bolívar State, Venezuela.

**Figure 9.**
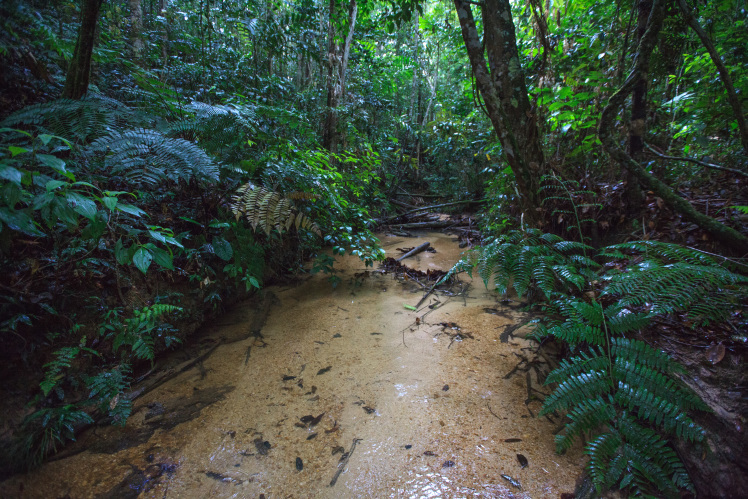
Photograph of collecting locality Biocor 12 in Bolívar State, Venezuela.

**Figure 10.**
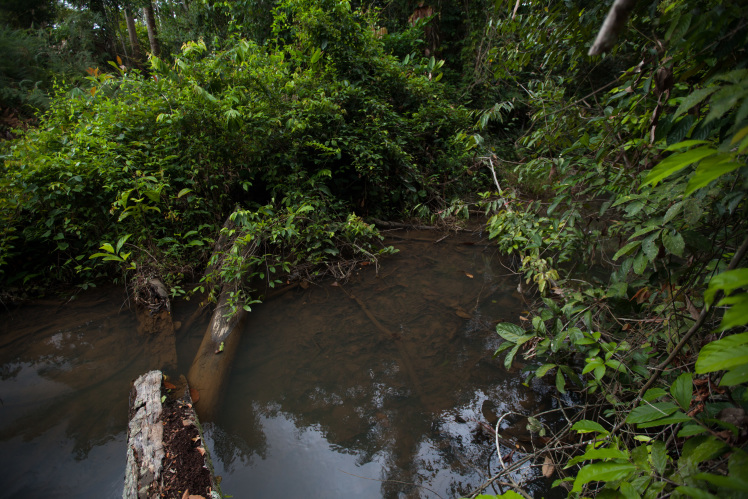
Photograph of collecting locality Biocor 13 in Bolívar State, Venezuela.

**Figure 11.**
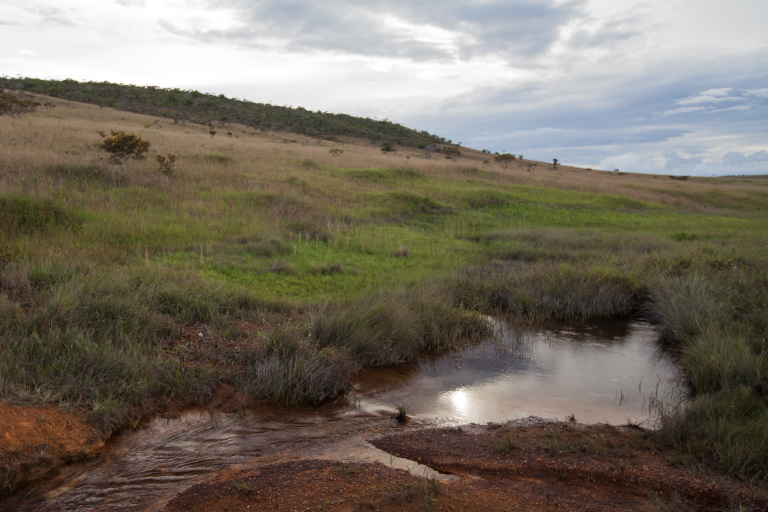
Photograph of collecting locality Biocor 14 in Bolívar State, Venezuela.

**Figure 12.**
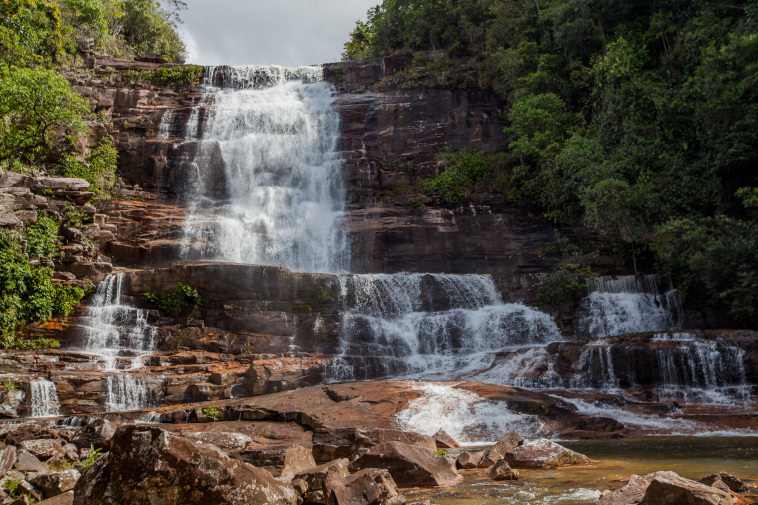
Photograph of collecting locality Biocor 15 in Bolívar State, Venezuela.

**Figure 13.**
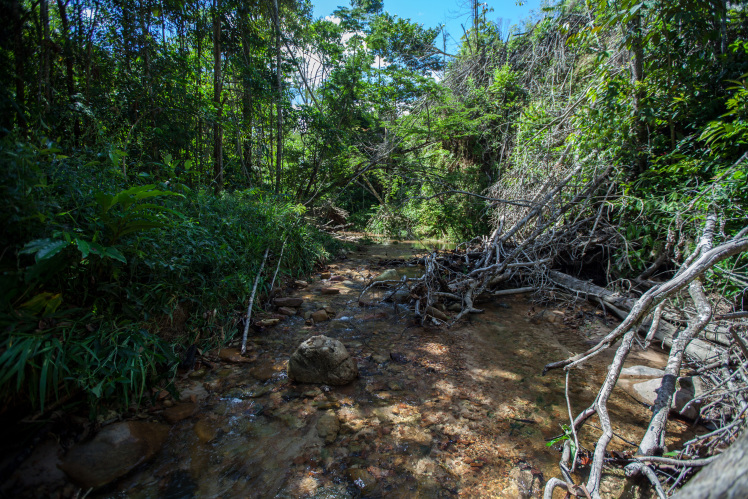
Photograph of collecting locality Biocor 16 in Bolívar State, Venezuela.

**Figure 14.**
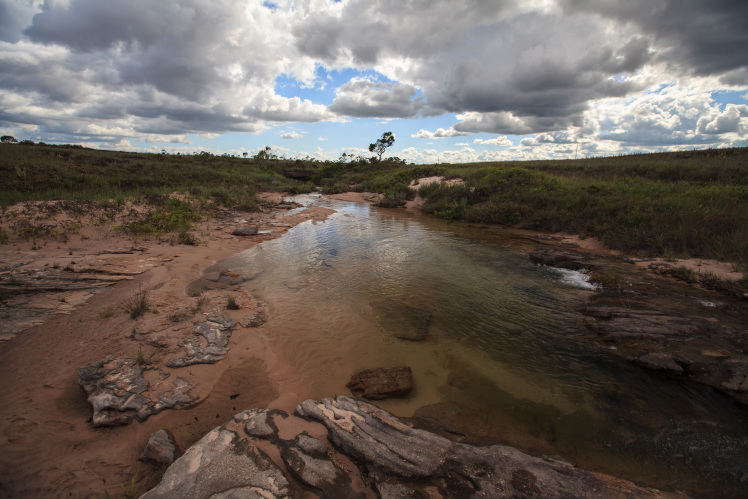
Photograph of collecting locality Biocor 17 in Bolívar State, Venezuela.

**Figure 15.**
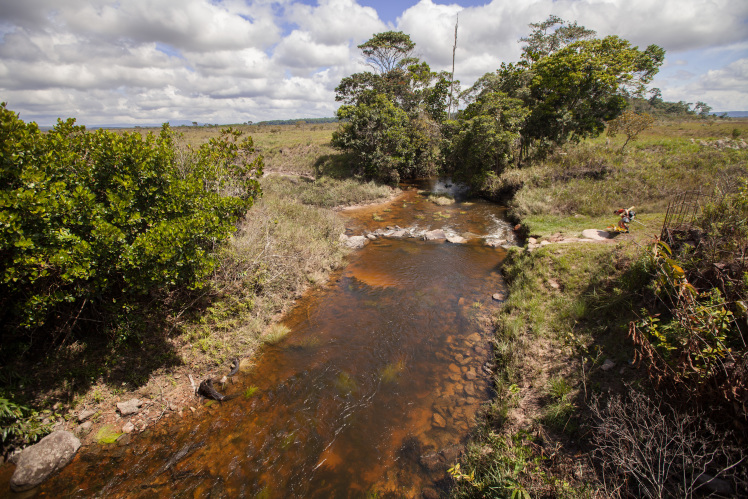
Photograph of collecting locality Biocor 18 in Bolívar State, Venezuela.

**Figure 16.**
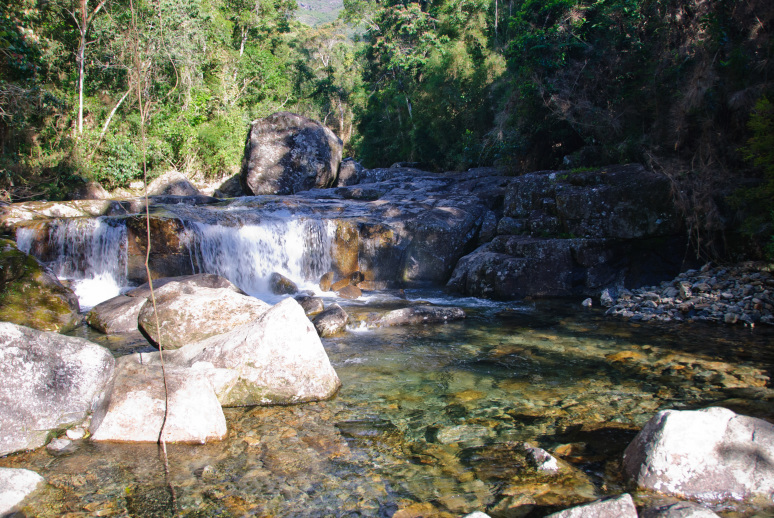
Photograph of collecting locality BR 7/2011 in Minas Gerais State, Brazil.

**Figure 17.**
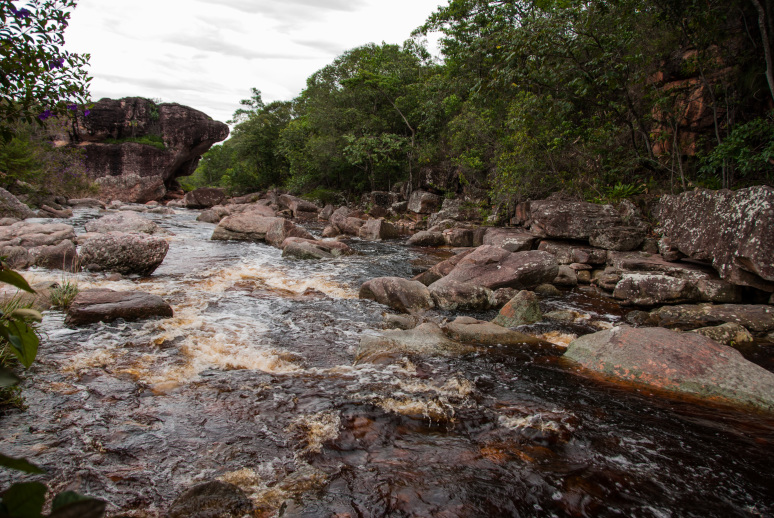
Photograph of collecting locality BR 8/2011 in Bahia State, Brazil.

**Figure 18.**
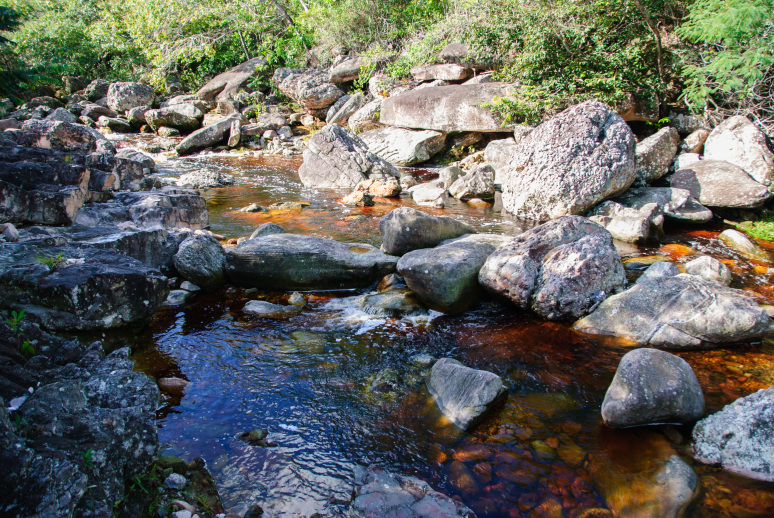
Photograph of collecting locality BR 11/2011 in Bahia State, Brazil.

Most of the material cited below was collected as part of the project "Biodiversity of river corridors of tropical forests: current status, impact of anthropogenic activity and the prospect of recovery" in Canaima National Park, Bolívar State, Venezuela. The park is part of the Venezuelan Guyana Region, has more than 30000 km^2^, and was considered a World Natural Heritage Site by UNESCO in 1994 (World Heritage Committee 1995), but the local fauna of water bugs has never been efficiently explored. Material examined is deposited in the Department of Biology and General Ecology, Faculty of Ecology and Environmental Sciences, Technical University in Zvolen, Zvolen, Slovakia (TUZVO). Main information about collecting localities is presented in Table 1. The known geographical distribution of each species is provided mainly according to ​Moreira (2016)​. New records are followed by an exclamation mark. Photographs showing some of the collecting localities are provided on Figs 1, 2, 3, 4, 5, 6, 7, 8, 9, 10, 11, 12, 13, 14, 15, 16, 17, 18.

## Taxon treatments

### Brachymetra lata

Shaw, 1933

#### Materials

- **Type status:** Other material. **Occurrence:** individualCount: 4; sex: 1 apterous male, 3 apterous females; **Taxon:** genus: Brachymetra; specificEpithet: lata; **Location:** continent: South America; country: Venezuela; stateProvince: Bolívar; county: Gran Sabana; locality: Canaima National Park; decimalLatitude: 4.91728; decimalLongitude: -61.09222; **Identification:** identifiedBy: Felipe F. F. Moreira; **Event:** year: 2012; month: 11; day: 29; fieldNumber: Biocor 03 kvalita; eventRemarks: M. Svitok col.; **Record Level:** type: PhysicalObject; institutionCode: TUZVO; basisOfRecord: PreservedSpecimen
- **Type status:** Other material. **Occurrence:** individualCount: 14; sex: 5 apterous males, 9 apterous females; **Taxon:** genus: Brachymetra; specificEpithet: lata; **Location:** continent: South America; country: Venezuela; stateProvince: Bolívar; county: Gran Sabana; locality: Canaima National Park; decimalLatitude: 5.03656; decimalLongitude: -61.07594; **Identification:** identifiedBy: Felipe F. F. Moreira; **Event:** year: 2012; month: 12; day: 2; fieldNumber: Biocor 11 kvalita; eventRemarks: M. Svitok col.; **Record Level:** type: PhysicalObject; institutionCode: TUZVO; basisOfRecord: PreservedSpecimen
- **Type status:** Other material. **Occurrence:** individualCount: 1; sex: macropterous male; **Taxon:** genus: Brachymetra; specificEpithet: lata; **Location:** continent: South America; country: Venezuela; stateProvince: Bolívar; county: Gran Sabana; locality: Canaima National Park; decimalLatitude: 5.03656; decimalLongitude: -61.07594; **Identification:** identifiedBy: Felipe F. F. Moreira; **Event:** year: 2012; month: 12; day: 2; fieldNumber: Biocor 11 kvantita; eventRemarks: M. Svitok col.; **Record Level:** type: PhysicalObject; institutionCode: TUZVO; basisOfRecord: PreservedSpecimen
- **Type status:** Other material. **Occurrence:** individualCount: 9; sex: 3 apterous males, 6 apterous females; **Taxon:** genus: Brachymetra; specificEpithet: lata; **Location:** continent: South America; country: Venezuela; stateProvince: Bolívar; county: Gran Sabana; decimalLatitude: 4.70389; decimalLongitude: -61.29169; **Identification:** identifiedBy: Felipe F. F. Moreira; **Event:** year: 2012; month: 12; day: 3; fieldNumber: Biocor 12 kvalita; eventRemarks: M. Svitok col.; **Record Level:** type: PhysicalObject; institutionCode: TUZVO; basisOfRecord: PreservedSpecimen

#### Distribution

Colombia, Venezuela!, Suriname, Brazil, Ecuador.

**Distribution in Venezuela**: Bolívar!.

### Cylindrostethus palmaris

Drake & Harris, 1934

#### Materials

- **Type status:** Other material. **Occurrence:** individualCount: 1; sex: apterous female; **Taxon:** genus: Cylindrostethus; specificEpithet: palmaris; **Location:** continent: South America; country: Venezuela; stateProvince: Bolívar; county: Gran Sabana; decimalLatitude: 4.70389; decimalLongitude: -61.29169; **Identification:** identifiedBy: Felipe F. F. Moreira; **Event:** year: 2012; month: 12; day: 3; fieldNumber: Biocor 12 kvalita; eventRemarks: M. Svitok col.; **Record Level:** type: PhysicalObject; institutionCode: TUZVO; basisOfRecord: PreservedSpecimen
- **Type status:** Other material. **Occurrence:** individualCount: 1; sex: apterous male; **Taxon:** genus: Cylindrostethus; specificEpithet: palmaris; **Location:** continent: South America; country: Venezuela; stateProvince: Bolívar; county: Gran Sabana; decimalLatitude: 4.70000; decimalLongitude: -61.33269; **Identification:** identifiedBy: Felipe F. F. Moreira; **Event:** year: 2012; month: 12; day: 3; fieldNumber: Biocor 13 kvalita; eventRemarks: M. Svitok col.; **Record Level:** type: PhysicalObject; institutionCode: TUZVO; basisOfRecord: PreservedSpecimen

#### Distribution

Colombia, Venezuela, Trinidad & Tobago, Guyana, Suriname, French Guiana, Brazil, Ecuador, Peru, Bolivia, Argentina.

**Distribution in Venezuela**: Aragua, Bolívar!.

### Halobatopsis platensis

(Berg, 1879)

#### Materials

- **Type status:** Other material. **Occurrence:** individualCount: 9; sex: 2 apterous males, 7 apterous females; **Taxon:** genus: Halobatopsis; specificEpithet: platensis; **Location:** continent: South America; country: Brazil; stateProvince: Bahia; municipality: Lençóis; locality: Rio Lençóis; decimalLatitude: -12.56014; decimalLongitude: -41.40442; **Identification:** identifiedBy: Higor D. D. Rodrigues; **Event:** year: 2011; month: 1; day: 12; fieldNumber: BR 8/2011; eventRemarks: M. Svitok col.; **Record Level:** type: PhysicalObject; institutionCode: TUZVO; basisOfRecord: PreservedSpecimen
- **Type status:** Other material. **Occurrence:** individualCount: 2; sex: 1 macropterous male, 1 macropterous female; **Taxon:** genus: Halobatopsis; specificEpithet: platensis; **Location:** continent: South America; country: Brazil; stateProvince: Bahia; municipality: Lençóis; locality: Rio Lençóis; decimalLatitude: -12.55894; decimalLongitude: -41.40489; **Identification:** identifiedBy: Felipe F. F. Moreira; **Event:** year: 2011; month: 1; day: 14; fieldNumber: BR 11/2011; eventRemarks: M. Svitok col.; **Record Level:** type: PhysicalObject; institutionCode: TUZVO; basisOfRecord: PreservedSpecimen

#### Distribution

Brazil, Argentina, Uruguay.

**Distribution in Brazil**: Piauí, Mato Grosso, Bahia, Goiás, Minas Gerais, Distrito Federal, Mato Grosso do Sul, São Paulo, Rio de Janeiro, Paraná, Rio Grande do Sul.

### Limnogonus hyalinus

(Fabricius, 1803)

#### Materials

- **Type status:** Other material. **Occurrence:** individualCount: 1; sex: macropterous male; **Taxon:** genus: Limnogonus; specificEpithet: hyalinus; **Location:** continent: South America; country: Venezuela; stateProvince: Bolívar; county: Gran Sabana; locality: Canaima National Park; decimalLatitude: 5.03508; decimalLongitude: -60.97569; **Identification:** identifiedBy: Felipe F. F. Moreira; **Event:** year: 2012; month: 12; day: 2; fieldNumber: Biocor 10 kvalita; eventRemarks: M. Svitok col.; **Record Level:** type: PhysicalObject; institutionCode: TUZVO; basisOfRecord: PreservedSpecimen

#### Distribution

Colombia, Venezuela!, Costa Rica, Trinidad & Tobago, Panama, Guyana, Suriname, French Guiana, Brazil, Ecuador, Bolivia.

**Distribution in Venezuela**: Bolívar!.

### Euvelia lata

Polhemus & Polhemus, 1984

#### Materials

- **Type status:** Other material. **Occurrence:** individualCount: 9; sex: 3 apterous males, 6 apterous females; **Taxon:** genus: Euvelia; specificEpithet: lata; **Location:** continent: South America; country: Peru; stateProvince: Loreto; county: Maynas; municipality: Iquitos; locality: Río Momón, Lores, 137 m a.s.l.; decimalLatitude: -3.51123; decimalLongitude: -73.40319; **Identification:** identifiedBy: Felipe F. F. Moreira; **Event:** year: 2013; month: 7; day: 19; eventRemarks: B. Reduciendo Klementová col.; **Record Level:** type: PhysicalObject; institutionCode: TUZVO; basisOfRecord: PreservedSpecimen

#### Distribution

Brazil, Peru!.

**Distribution in Peru**: Loreto!.

### Microvelia pulchella

Westwood, 1834

#### Materials

- **Type status:** Other material. **Occurrence:** individualCount: 11; sex: 2 apterous males, 3 macropterous males, 6 apterous females; **Taxon:** genus: Microvelia; specificEpithet: pulchella; **Location:** continent: South America; country: Peru; stateProvince: Arequipa; county: La Unión; locality: Cotahuasi Canyon, Laguna Chaquicocha, 2595 m a.s.l.; decimalLatitude: -15.20453; decimalLongitude: -72.89195; **Identification:** identifiedBy: Felipe F. F. Moreira; **Event:** year: 2013; month: 5; day: 2; eventRemarks: B. Reduciendo Klementová col.; **Record Level:** type: PhysicalObject; institutionCode: TUZVO; basisOfRecord: PreservedSpecimen

#### Distribution

Canada, USA, Mexico, Bahamas, Cuba, Dominican Republic, Puerto Rico, U.S. Virgin Islands, Guatemala, Cayman Islands, Jamaica, Anguilla, St. Martin, Saba, St. Kitts & Nevis, Guadeloupe, Martinique, Aruba, Colombia, St. Vincent & Grenadines, Barbados, Curaçao, Klein Curaçao, Bonaire, Klein Bonaire, Grenada, Venezuela, Costa Rica, Trinidad & Tobago, Panama, Brazil, Ecuador, Peru, Argentina.

**Distribution in Peru**: Arequipa! [previously recorded from the country without details by Drake and Plaumann (1955)​].

### Rhagovelia elegans

Uhler, 1894

#### Materials

- **Type status:** Other material. **Occurrence:** individualCount: 25; sex: 25 apterous females; **Taxon:** genus: Rhagovelia; specificEpithet: elegans; **Location:** continent: South America; country: Venezuela; stateProvince: Bolívar; county: Gran Sabana; locality: Canaima National Park; decimalLatitude: 5.03656; decimalLongitude: -61.07594; **Identification:** identifiedBy: Felipe F. F. Moreira; **Event:** year: 2012; month: 12; day: 2; fieldNumber: Biocor 11 kvalita; eventRemarks: M. Svitok; **Record Level:** type: PhysicalObject; institutionCode: TUZVO; basisOfRecord: PreservedSpecimen

#### Distribution

Hispaniola Island, St. Kitts & Nevis, Dominica, Martinique, St. Lucia, St. Vincent & Grenadines, Colombia, Grenada, Venezuela, Costa Rica, Trinidad & Tobago, Panama, Brazil, Ecuador.

**Distribution in Venezuela**: Carabobo, Monagas, Bolívar!.

### Rhagovelia evidis

Bacon, 1948

#### Materials

- **Type status:** Other material. **Occurrence:** individualCount: 5; sex: 5 apterous females; **Taxon:** genus: Rhagovelia; specificEpithet: evidis; **Location:** continent: South America; country: Venezuela; stateProvince: Bolívar; county: Gran Sabana; decimalLatitude: 4.70389; decimalLongitude: -61.29169; **Identification:** identifiedBy: Felipe F. F. Moreira; **Event:** year: 2012; month: 12; day: 3; fieldNumber: Biocor 12 kvalita; eventRemarks: M. Svitok; **Record Level:** type: PhysicalObject; institutionCode: TUZVO; basisOfRecord: PreservedSpecimen

#### Distribution

Venezuela!, Brazil, Peru.

**Distribution in Venezuela**: Bolívar!.

### Rhagovelia tenuipes

Champion, 1898

#### Materials

- **Type status:** Other material. **Occurrence:** individualCount: 3; sex: 3 apterous females; **Taxon:** genus: Rhagovelia; specificEpithet: tenuipes; **Location:** continent: South America; country: Venezuela; stateProvince: Bolívar; county: Gran Sabana; locality: Canaima National Park; decimalLatitude: 5.67315; decimalLongitude: -61.40467; **Identification:** identifiedBy: Felipe F. F. Moreira; **Event:** year: 2012; month: 11; day: 28; fieldNumber: Biocor 01 kvalita; eventRemarks: M. Svitok; **Record Level:** type: PhysicalObject; institutionCode: TUZVO; basisOfRecord: PreservedSpecimen
- **Type status:** Other material. **Occurrence:** individualCount: 2; sex: 1 apterous male, 1 apterous female; **Taxon:** genus: Rhagovelia; specificEpithet: tenuipes; **Location:** continent: South America; country: Venezuela; stateProvince: Bolívar; county: Gran Sabana; locality: Canaima National Park; decimalLatitude: 4.91728; decimalLongitude: -61.09222; **Identification:** identifiedBy: Felipe F. F. Moreira; **Event:** year: 2012; month: 11; day: 29; fieldNumber: Biocor 03 kvalita; eventRemarks: M. Svitok; **Record Level:** type: PhysicalObject; institutionCode: TUZVO; basisOfRecord: PreservedSpecimen
- **Type status:** Other material. **Occurrence:** individualCount: 19; sex: 10 apterous males, 9 apterous females; **Taxon:** genus: Rhagovelia; specificEpithet: tenuipes; **Location:** continent: South America; country: Venezuela; stateProvince: Bolívar; county: Gran Sabana; locality: Canaima National Park; decimalLatitude: 4.89658; decimalLongitude: -61.09136; **Identification:** identifiedBy: Felipe F. F. Moreira; **Event:** year: 2012; month: 11; day: 29; fieldNumber: Biocor 04 kvalita; eventRemarks: M. Svitok; **Record Level:** type: PhysicalObject; institutionCode: TUZVO; basisOfRecord: PreservedSpecimen
- **Type status:** Other material. **Occurrence:** individualCount: 1; sex: 1 apterous female; **Taxon:** genus: Rhagovelia; specificEpithet: tenuipes; **Location:** continent: South America; country: Venezuela; stateProvince: Bolívar; county: Gran Sabana; locality: Canaima National Park; decimalLatitude: 4.89658; decimalLongitude: -61.09136; **Identification:** identifiedBy: Felipe F. F. Moreira; **Event:** year: 2012; month: 11; day: 29; fieldNumber: Biocor 04 kvantita; eventRemarks: M. Svitok; **Record Level:** type: PhysicalObject; institutionCode: TUZVO; basisOfRecord: PreservedSpecimen
- **Type status:** Other material. **Occurrence:** individualCount: 1; sex: 1 apterous male; **Taxon:** genus: Rhagovelia; specificEpithet: tenuipes; **Location:** continent: South America; country: Venezuela; stateProvince: Bolívar; county: Gran Sabana; locality: Canaima National Park; decimalLatitude: 5.15958; decimalLongitude: -61.10431; **Identification:** identifiedBy: Felipe F. F. Moreira; **Event:** year: 2012; month: 12; day: 4; fieldNumber: Biocor 15 kvalita; eventRemarks: M. Svitok; **Record Level:** type: PhysicalObject; institutionCode: TUZVO; basisOfRecord: PreservedSpecimen
- **Type status:** Other material. **Occurrence:** individualCount: 1; sex: 1 apterous male; **Taxon:** genus: Rhagovelia; specificEpithet: tenuipes; **Location:** continent: South America; country: Venezuela; stateProvince: Bolívar; county: Gran Sabana; locality: Canaima National Park; decimalLatitude: 5.28636; decimalLongitude: -61.11033; **Identification:** identifiedBy: Felipe F. F. Moreira; **Event:** year: 2012; month: 12; day: 4; fieldNumber: Biocor 16 kvalita; eventRemarks: M. Svitok; **Record Level:** type: PhysicalObject; institutionCode: TUZVO; basisOfRecord: PreservedSpecimen
- **Type status:** Other material. **Occurrence:** individualCount: 1; sex: 1 apterous female; **Taxon:** genus: Rhagovelia; specificEpithet: tenuipes; **Location:** continent: South America; country: Venezuela; stateProvince: Bolívar; county: Gran Sabana; locality: left side tributary below Salto del Danto, 1100 m a.s.l.; decimalLatitude: 5.96433; decimalLongitude: -61.38264; **Identification:** identifiedBy: Felipe F. F. Moreira; **Event:** year: 2011; month: 11; day: 19; fieldNumber: VEN 4/2011; eventRemarks: M. Svitok; **Record Level:** type: PhysicalObject; institutionCode: TUZVO; basisOfRecord: PreservedSpecimen

#### Distribution

Mexico, Cayman Islands, Belize, Guatemala, Honduras, Nicaragua, Colombia, Venezuela, Costa Rica, Trinidad & Tobago, Brazil, Ecuador, Peru.

**Distribution in Venezuela**: Vargas, Carabobo, Monagas, Bolívar!.

### Rhagovelia triangula

Drake, 1953

#### Materials

- **Type status:** Other material. **Occurrence:** individualCount: 8; sex: 7 apterous males, 1 apterous female; **Taxon:** genus: Rhagovelia; specificEpithet: triangula; **Location:** continent: South America; country: Brazil; stateProvince: Minas Gerais; municipality: Alto Caparaó; locality: Rio Caparaó; decimalLatitude: -20.43300; decimalLongitude: -41.86672; **Identification:** identifiedBy: Felipe F. F. Moreira; **Event:** year: 2011; month: 1; day: 7; eventRemarks: M. Svitok; **Record Level:** type: PhysicalObject; institutionCode: TUZVO; basisOfRecord: PreservedSpecimen

#### Distribution

Brazil.

**Distribution in Brazil**: Minas Gerais, Espírito Santo, São Paulo, Rio de Janeiro.

### Rhagovelia yanomamo

Polhemus, 1997

#### Materials

- **Type status:** Other material. **Occurrence:** individualCount: 14; sex: 5 apterous males, 1 macropterous male, 8 apterous females; **Taxon:** genus: Rhagovelia; specificEpithet: yanomamo; **Location:** continent: South America; country: Venezuela; stateProvince: Bolívar; county: Gran Sabana; locality: Canaima National Park; decimalLatitude: 4.86188; decimalLongitude: -61.10061; **Identification:** identifiedBy: Felipe F. F. Moreira; **Event:** year: 2012; month: 11; day: 30; fieldNumber: Biocor 06 kvantita; eventRemarks: M. Svitok; **Record Level:** type: PhysicalObject; institutionCode: TUZVO; basisOfRecord: PreservedSpecimen

#### Distribution

Venezuela, Guyana, Brazil.

**Distribution in Venezuela**: Bolívar, Amazonas.

### Tenagobia (Romanogobia) peruana

Egbert, 1949

#### Materials

- **Type status:** Other material. **Taxon:** genus: Tenagobia; subgenus: Romanogobia; specificEpithet: peruana; **Location:** continent: South America; country: Venezuela; stateProvince: Bolívar; county: Gran Sabana; locality: Canaima National Park; decimalLatitude: 5.57708; decimalLongitude: -61.31242; **Identification:** identifiedBy: Julianna F. Barbosa; **Event:** year: 2012; month: 12; day: 5; fieldNumber: Biocor 18 kvalita; eventRemarks: M. Svitok; **Record Level:** type: PhysicalObject; institutionCode: TUZVO; basisOfRecord: PreservedSpecimen

#### Distribution

Venezuela!, Peru.

**Distribution in Venezuela**: Bolívar!.

### Ambrysus montandoni

La Rivers, 1963

#### Materials

- **Type status:** Other material. **Occurrence:** individualCount: 1; sex: brachypterous female; **Taxon:** genus: Ambrysus; specificEpithet: montandoni; **Location:** continent: South America; country: Venezuela; stateProvince: Bolívar; county: Gran Sabana; locality: Canaima National Park; decimalLatitude: 5.21005; decimalLongitude: -61.09400; **Identification:** identifiedBy: Higor D. D. Rodrigues; **Event:** year: 2012; month: 12; day: 4; fieldNumber: Biocor 17 kvalita; eventRemarks: M. Svitok col.; **Record Level:** type: PhysicalObject; institutionCode: TUZVO; basisOfRecord: PreservedSpecimen

#### Distribution

Venezuela, Brazil.

**Distribution in Venezuela**: Bolívar, Amazonas.

### Ambrysus stali

La Rivers, 1962

#### Materials

- **Type status:** Other material. **Occurrence:** individualCount: 1; sex: macropterous male; **Taxon:** genus: Ambrysus; specificEpithet: stali; **Location:** continent: South America; country: Venezuela; stateProvince: Bolívar; county: Gran Sabana; locality: Canaima National Park; decimalLatitude: 4.94219; decimalLongitude: -61.08764; **Identification:** identifiedBy: Higor D. D. Rodrigues; **Event:** year: 2012; month: 12; day: 1; fieldNumber: Biocor 08 kvalita; eventRemarks: M. Svitok col.; **Record Level:** type: PhysicalObject; institutionCode: TUZVO; basisOfRecord: PreservedSpecimen

#### Distribution

Venezuela, Trinidad & Tobago, Guyana, Suriname, French Guiana, Brazil. According to Sites and Reynoso-Velasco (2015), specimens from Argentina (López-Ruf 1987, López-Ruf 2007, Mazzuconi et al. 2009​) are probably not conspecific with *A. stali*.

**Distribution in Venezuela**: Bolívar!, Amazonas.

### Limnocoris brasiliensis

De Carlo, 1941

#### Materials

- **Type status:** Other material. **Occurrence:** individualCount: 3; sex: 1 macropterous male, 2 brachypterous females; **Taxon:** genus: Limnocoris; specificEpithet: brasiliensis; **Location:** continent: South America; country: Brazil; stateProvince: Minas Gerais; municipality: Alto Caparaó; locality: Vale Verde; decimalLatitude: -20.42000; decimalLongitude: -41.84486; **Identification:** identifiedBy: Higor D. D. Rodrigues; **Event:** year: 2011; month: 1; day: 9; fieldNumber: BR 7/2011; eventRemarks: M. Svitok col.; **Record Level:** type: PhysicalObject; institutionCode: TUZVO; basisOfRecord: PreservedSpecimen
- **Type status:** Other material. **Occurrence:** individualCount: 3; sex: 1 brachypterous male, 1 macropterous male, 1 brachypterous female; **Taxon:** genus: Limnocoris; specificEpithet: brasiliensis; **Location:** continent: South America; country: Brazil; stateProvince: Minas Gerais; municipality: Alto Caparaó; locality: Rio Caparaó; decimalLatitude: -20.43300; decimalLongitude: -41.86672; **Identification:** identifiedBy: Higor D. D. Rodrigues; **Event:** year: 2011; month: 1; day: 7; eventRemarks: M. Svitok col.; **Record Level:** type: PhysicalObject; institutionCode: TUZVO; basisOfRecord: PreservedSpecimen

#### Distribution

Brazil.

**Distribution in Brazil**: Minas Gerais, São Paulo, Rio de Janeiro.

### Limnocoris burmeisteri

De Carlo, 1967

#### Materials

- **Type status:** Other material. **Occurrence:** individualCount: 4; sex: 2 brachypterous males, 2 brachypterous females; **Taxon:** genus: Limnocoris; specificEpithet: burmeisteri; **Location:** continent: South America; country: Venezuela; stateProvince: Bolívar; county: Gran Sabana; locality: Canaima National Park; decimalLatitude: 5.67315; decimalLongitude: -61.40467; **Identification:** identifiedBy: Higor D. D. Rodrigues; **Event:** year: 2012; month: 11; day: 28; fieldNumber: Biocor 01 kvalita; eventRemarks: M. Svitok col.; **Record Level:** type: PhysicalObject; institutionCode: TUZVO; basisOfRecord: PreservedSpecimen
- **Type status:** Other material. **Occurrence:** individualCount: 2; sex: 2 brachypterous females; **Taxon:** genus: Limnocoris; specificEpithet: burmeisteri; **Location:** continent: South America; country: Venezuela; stateProvince: Bolívar; county: Gran Sabana; locality: Canaima National Park; decimalLatitude: 5.65003; decimalLongitude: -61.39197; **Identification:** identifiedBy: Higor D. D. Rodrigues; **Event:** year: 2012; month: 11; day: 28; fieldNumber: Biocor 02 kvalita; eventRemarks: M. Svitok col.; **Record Level:** type: PhysicalObject; institutionCode: TUZVO; basisOfRecord: PreservedSpecimen
- **Type status:** Other material. **Occurrence:** individualCount: 1; sex: 1 brachypterous male; **Taxon:** genus: Limnocoris; specificEpithet: burmeisteri; **Location:** continent: South America; country: Venezuela; stateProvince: Bolívar; county: Gran Sabana; locality: Canaima National Park; decimalLatitude: 4.91728; decimalLongitude: -61.09222; **Identification:** identifiedBy: Higor D. D. Rodrigues; **Event:** year: 2012; month: 11; day: 29; fieldNumber: Biocor 03 kvalita; eventRemarks: M. Svitok col.; **Record Level:** type: PhysicalObject; institutionCode: TUZVO; basisOfRecord: PreservedSpecimen
- **Type status:** Other material. **Occurrence:** individualCount: 2; sex: 1 brachypterous male, 1 brachypterous female; **Taxon:** genus: Limnocoris; specificEpithet: burmeisteri; **Location:** continent: South America; country: Venezuela; stateProvince: Bolívar; county: Gran Sabana; locality: Canaima National Park; decimalLatitude: 4.89658; decimalLongitude: -61.09136; **Identification:** identifiedBy: Higor D. D. Rodrigues; **Event:** year: 2012; month: 11; day: 29; fieldNumber: Biocor 04 kvalita; eventRemarks: M. Svitok col.; **Record Level:** type: PhysicalObject; institutionCode: TUZVO; basisOfRecord: PreservedSpecimen
- **Type status:** Other material. **Occurrence:** individualCount: 1; sex: 1 brachypterous female; **Taxon:** genus: Limnocoris; specificEpithet: burmeisteri; **Location:** continent: South America; country: Venezuela; stateProvince: Bolívar; county: Gran Sabana; locality: Canaima National Park; decimalLatitude: 4.89658; decimalLongitude: -61.09136; **Identification:** identifiedBy: Higor D. D. Rodrigues; **Event:** year: 2012; month: 11; day: 29; fieldNumber: Biocor 04 kvantita; eventRemarks: M. Svitok col.; **Record Level:** type: PhysicalObject; institutionCode: TUZVO; basisOfRecord: PreservedSpecimen
- **Type status:** Other material. **Occurrence:** individualCount: 1; sex: 1 brachypterous male; **Taxon:** genus: Limnocoris; specificEpithet: burmeisteri; **Location:** continent: South America; country: Venezuela; stateProvince: Bolívar; county: Gran Sabana; locality: Canaima National Park; decimalLatitude: 4.86188; decimalLongitude: -61.10061; **Identification:** identifiedBy: Higor D. D. Rodrigues; **Event:** year: 2012; month: 11; day: 30; fieldNumber: Biocor 06 kvantita; eventRemarks: M. Svitok col.; **Record Level:** type: PhysicalObject; institutionCode: TUZVO; basisOfRecord: PreservedSpecimen
- **Type status:** Other material. **Occurrence:** individualCount: 10; sex: 9 brachypterous males, 1 brachypterous female; **Taxon:** genus: Limnocoris; specificEpithet: burmeisteri; **Location:** continent: South America; country: Venezuela; stateProvince: Bolívar; county: Gran Sabana; locality: Canaima National Park; decimalLatitude: 5.03656; decimalLongitude: -61.07594; **Identification:** identifiedBy: Higor D. D. Rodrigues; **Event:** year: 2012; month: 12; day: 2; fieldNumber: Biocor 11 kvalita; eventRemarks: M. Svitok col.; **Record Level:** type: PhysicalObject; institutionCode: TUZVO; basisOfRecord: PreservedSpecimen
- **Type status:** Other material. **Occurrence:** individualCount: 2; sex: 1 brachypterous male, 1 brachypterous female; **Taxon:** genus: Limnocoris; specificEpithet: burmeisteri; **Location:** continent: South America; country: Venezuela; stateProvince: Bolívar; county: Gran Sabana; locality: Canaima National Park; decimalLatitude: 5.03656; decimalLongitude: -61.07594; **Identification:** identifiedBy: Higor D. D. Rodrigues; **Event:** year: 2012; month: 12; day: 2; fieldNumber: Biocor 11 kvantita; eventRemarks: M. Svitok col.; **Record Level:** type: PhysicalObject; institutionCode: TUZVO; basisOfRecord: PreservedSpecimen
- **Type status:** Other material. **Occurrence:** individualCount: 4; sex: 3 brachypterous males, 1 brachypterous female; **Taxon:** genus: Limnocoris; specificEpithet: burmeisteri; **Location:** continent: South America; country: Venezuela; stateProvince: Bolívar; county: Gran Sabana; decimalLatitude: 4.70389; decimalLongitude: -61.29169; **Identification:** identifiedBy: Higor D. D. Rodrigues; **Event:** year: 2012; month: 12; day: 3; fieldNumber: Biocor 12 kvalita; eventRemarks: M. Svitok col.; **Record Level:** type: PhysicalObject; institutionCode: TUZVO; basisOfRecord: PreservedSpecimen
- **Type status:** Other material. **Occurrence:** individualCount: 5; sex: 4 brachypterous males, 1 brachypterous female; **Taxon:** genus: Limnocoris; specificEpithet: burmeisteri; **Location:** continent: South America; country: Venezuela; stateProvince: Bolívar; county: Gran Sabana; decimalLatitude: 4.70000; decimalLongitude: -61.33269; **Identification:** identifiedBy: Higor D. D. Rodrigues; **Event:** year: 2012; month: 12; day: 3; fieldNumber: Biocor 13 kvalita; eventRemarks: M. Svitok col.; **Record Level:** type: PhysicalObject; institutionCode: TUZVO; basisOfRecord: PreservedSpecimen
- **Type status:** Other material. **Occurrence:** individualCount: 14; sex: 5 brachypterous males, 9 brachypterous females; **Taxon:** genus: Limnocoris; specificEpithet: burmeisteri; **Location:** continent: South America; country: Venezuela; stateProvince: Bolívar; county: Gran Sabana; decimalLatitude: 4.70000; decimalLongitude: -61.33269; **Identification:** identifiedBy: Higor D. D. Rodrigues; **Event:** year: 2012; month: 12; day: 3; fieldNumber: Biocor 13 kvantita; eventRemarks: M. Svitok col.; **Record Level:** type: PhysicalObject; institutionCode: TUZVO; basisOfRecord: PreservedSpecimen
- **Type status:** Other material. **Occurrence:** individualCount: 8; sex: 4 brachypterous males, 4 brachypterous females; **Taxon:** genus: Limnocoris; specificEpithet: burmeisteri; **Location:** continent: South America; country: Venezuela; stateProvince: Bolívar; county: Gran Sabana; locality: Canaima National Park; decimalLatitude: 4.63033; decimalLongitude: -61.32733; **Identification:** identifiedBy: Higor D. D. Rodrigues; **Event:** year: 2012; month: 12; day: 3; fieldNumber: Biocor 14 kvalita; eventRemarks: M. Svitok col.; **Record Level:** type: PhysicalObject; institutionCode: TUZVO; basisOfRecord: PreservedSpecimen
- **Type status:** Other material. **Occurrence:** individualCount: 1; sex: 1 brachypterous male; **Taxon:** genus: Limnocoris; specificEpithet: burmeisteri; **Location:** continent: South America; country: Venezuela; stateProvince: Bolívar; county: Gran Sabana; locality: Canaima National Park; decimalLatitude: 4.63033; decimalLongitude: -61.32733; **Identification:** identifiedBy: Higor D. D. Rodrigues; **Event:** year: 2012; month: 12; day: 3; fieldNumber: Biocor 14 kvantita; eventRemarks: M. Svitok col.; **Record Level:** type: PhysicalObject; institutionCode: TUZVO; basisOfRecord: PreservedSpecimen
- **Type status:** Other material. **Occurrence:** individualCount: 5; sex: 3 brachypterous males, 1 macropterous male, 1 brachypterous female; **Taxon:** genus: Limnocoris; specificEpithet: burmeisteri; **Location:** continent: South America; country: Venezuela; stateProvince: Bolívar; county: Gran Sabana; locality: Canaima National Park; decimalLatitude: 5.15958; decimalLongitude: -61.10431; **Identification:** identifiedBy: Higor D. D. Rodrigues; **Event:** year: 2012; month: 12; day: 4; fieldNumber: Biocor 15 kvalita; eventRemarks: M. Svitok col.; **Record Level:** type: PhysicalObject; institutionCode: TUZVO; basisOfRecord: PreservedSpecimen
- **Type status:** Other material. **Occurrence:** individualCount: 3; sex: 3 brachypterous males; **Taxon:** genus: Limnocoris; specificEpithet: burmeisteri; **Location:** continent: South America; country: Venezuela; stateProvince: Bolívar; county: Gran Sabana; locality: Canaima National Park; decimalLatitude: 5.28636; decimalLongitude: -61.11033; **Identification:** identifiedBy: Higor D. D. Rodrigues; **Event:** year: 2012; month: 12; day: 4; fieldNumber: Biocor 16 kvantita; eventRemarks: M. Svitok col.; **Record Level:** type: PhysicalObject; institutionCode: TUZVO; basisOfRecord: PreservedSpecimen
- **Type status:** Other material. **Occurrence:** individualCount: 7; sex: 2 brachypterous males, 2 macropterous males, 3 brachypterous females; **Taxon:** genus: Limnocoris; specificEpithet: burmeisteri; **Location:** continent: South America; country: Venezuela; stateProvince: Bolívar; county: Gran Sabana; locality: Canaima National Park; decimalLatitude: 5.21005; decimalLongitude: -61.09400; **Identification:** identifiedBy: Higor D. D. Rodrigues; **Event:** year: 2012; month: 12; day: 4; fieldNumber: Biocor 17 kvalita; eventRemarks: M. Svitok col.; **Record Level:** type: PhysicalObject; institutionCode: TUZVO; basisOfRecord: PreservedSpecimen
- **Type status:** Other material. **Occurrence:** individualCount: 1; sex: 1 brachypterous male; **Taxon:** genus: Limnocoris; specificEpithet: burmeisteri; **Location:** continent: South America; country: Venezuela; stateProvince: Bolívar; county: Gran Sabana; locality: Canaima National Park; decimalLatitude: 5.21005; decimalLongitude: -61.09400; **Identification:** identifiedBy: Higor D. D. Rodrigues; **Event:** year: 2012; month: 12; day: 4; fieldNumber: Biocor 17 kvantita; eventRemarks: M. Svitok col.; **Record Level:** type: PhysicalObject; institutionCode: TUZVO; basisOfRecord: PreservedSpecimen
- **Type status:** Other material. **Occurrence:** individualCount: 8; sex: 5 brachypterous males, 1 macropterous male, 2 brachypterous females; **Taxon:** genus: Limnocoris; specificEpithet: burmeisteri; **Location:** continent: South America; country: Venezuela; stateProvince: Bolívar; county: Gran Sabana; locality: Canaima National Park; decimalLatitude: 5.57708; decimalLongitude: -61.31242; **Identification:** identifiedBy: Higor D. D. Rodrigues; **Event:** year: 2012; month: 12; day: 5; fieldNumber: Biocor 18 kvalita; eventRemarks: M. Svitok col.; **Record Level:** type: PhysicalObject; institutionCode: TUZVO; basisOfRecord: PreservedSpecimen
- **Type status:** Other material. **Occurrence:** individualCount: 2; sex: 1 brachypterous male, 1 brachypterous female; **Taxon:** genus: Limnocoris; specificEpithet: burmeisteri; **Location:** continent: South America; country: Venezuela; stateProvince: Bolívar; county: Gran Sabana; locality: Canaima National Park; decimalLatitude: 5.57708; decimalLongitude: -61.31242; **Identification:** identifiedBy: Higor D. D. Rodrigues; **Event:** year: 2012; month: 12; day: 5; fieldNumber: Biocor 18 kvantita; eventRemarks: M. Svitok col.; **Record Level:** type: PhysicalObject; institutionCode: TUZVO; basisOfRecord: PreservedSpecimen

#### Distribution

Venezuela!, Suriname, Brazil.

**Distribution in Venezuela**: Bolívar!.

### Limnocoris fittkaui fittkaui

De Carlo, 1967

#### Materials

- **Type status:** Other material. **Occurrence:** individualCount: 23; sex: 9 macropterous males, 14 macropterous females; **Taxon:** genus: Limnocoris; specificEpithet: fittkaui; infraspecificEpithet: fittkaui; **Location:** continent: South America; country: Venezuela; stateProvince: Bolívar; county: Gran Sabana; locality: Canaima National Park; decimalLatitude: 4.63033; decimalLongitude: -61.32733; **Identification:** identifiedBy: Higor D. D. Rodrigues; **Event:** year: 2012; month: 12; day: 3; fieldNumber: Biocor 14 kvalita; eventRemarks: M. Svitok col.; **Record Level:** type: PhysicalObject; institutionCode: TUZVO; basisOfRecord: PreservedSpecimen
- **Type status:** Other material. **Occurrence:** individualCount: 9; sex: 7 macropterous males, 2 macropterous females; **Taxon:** genus: Limnocoris; specificEpithet: fittkaui; infraspecificEpithet: fittkaui; **Location:** continent: South America; country: Venezuela; stateProvince: Bolívar; county: Gran Sabana; locality: Canaima National Park; decimalLatitude: 4.63033; decimalLongitude: -61.32733; **Identification:** identifiedBy: Higor D. D. Rodrigues; **Event:** year: 2012; month: 12; day: 3; fieldNumber: Biocor 14 kvantita; eventRemarks: M. Svitok col.; **Record Level:** type: PhysicalObject; institutionCode: TUZVO; basisOfRecord: PreservedSpecimen
- **Type status:** Other material. **Occurrence:** individualCount: 1; sex: 1 macropterous male; **Taxon:** genus: Limnocoris; specificEpithet: fittkaui; infraspecificEpithet: fittkaui; **Location:** continent: South America; country: Venezuela; stateProvince: Bolívar; county: Gran Sabana; locality: Canaima National Park; decimalLatitude: 5.21005; decimalLongitude: -61.094; **Identification:** identifiedBy: Higor D. D. Rodrigues; **Event:** year: 2012; month: 12; day: 4; fieldNumber: Biocor 17 kvalita; eventRemarks: M. Svitok col.; **Record Level:** type: PhysicalObject; institutionCode: TUZVO; basisOfRecord: PreservedSpecimen

#### Distribution

Colombia, Venezuela!, Brazil.

**Distribution in Venezuela:** Bolívar!.

### Limnocoris volxemi

(Lethierry, 1877)

#### Materials

- **Type status:** Other material. **Occurrence:** individualCount: 1; sex: macropterous male; **Taxon:** genus: Limnocoris; specificEpithet: volxemi; **Location:** continent: South America; country: Brazil; stateProvince: Minas Gerais; municipality: Alto Caparaó; locality: Rio Caparaó; decimalLatitude: -20.43300; decimalLongitude: -41.86672; **Identification:** identifiedBy: Higor D. D. Rodrigues; **Event:** year: 2011; month: 1; day: 7; eventRemarks: M. Svitok col.; **Record Level:** type: PhysicalObject; institutionCode: TUZVO; basisOfRecord: PreservedSpecimen

#### Distribution

Brazil.

**Distribution in Brazil**: Mato Grosso, Minas Gerais, Paraná, Santa Catarina.

### Placomerus micans

La Rivers, 1956

#### Materials

- **Type status:** Other material. **Occurrence:** individualCount: 1; sex: 1 macropterous male, hindwing brachypterous; **Taxon:** genus: Placomerus; specificEpithet: micans; **Location:** continent: South America; country: Venezuela; stateProvince: Bolívar; county: Gran Sabana; locality: Canaima National Park; decimalLatitude: 5.67315; decimalLongitude: -61.40467; **Identification:** identifiedBy: Higor D. D. Rodrigues; **Event:** year: 2012; month: 11; day: 28; fieldNumber: Biocor 01 kvalita; eventRemarks: M. Svitok; **Record Level:** type: PhysicalObject; institutionCode: TUZVO; basisOfRecord: PreservedSpecimen

#### Distribution

Venezuela!, Brazil, Bolivia, Paraguay, Argentina.

**Distribution in Venezuela**: Bolívar!.

### Martarega bentoi

Truxal, 1949

#### Materials

- **Type status:** Other material. **Taxon:** genus: Martarega; specificEpithet: bentoi; **Location:** continent: South America; country: Brazil; stateProvince: Bahia; municipality: Lençóis; locality: Rio Lençóis; decimalLatitude: -12.55894; decimalLongitude: -41.40489; **Identification:** identifiedBy: Juliana F. Barbosa; **Event:** year: 2011; month: 1; day: 14; fieldNumber: BR 11/2011; eventRemarks: M. Svitok col.; **Record Level:** type: PhysicalObject; institutionCode: TUZVO; basisOfRecord: PreservedSpecimen

#### Distribution

Brazil, Argentina.

**Distribution in Brazil**: Piauí, Pernambuco, Mato Grosso, Bahia!, Minas Gerais, Rio de Janeiro.

### Martarega gonostyla

Truxal, 1949

#### Materials

- **Type status:** Other material. **Taxon:** genus: Martarega; specificEpithet: gonostyla; **Location:** continent: South America; country: Venezuela; stateProvince: Bolívar; county: Gran Sabana; locality: Canaima National Park; decimalLatitude: 4.86188; decimalLongitude: -61.10061; **Identification:** identifiedBy: Juliana F. Barbosa; **Event:** year: 2012; month: 11; day: 30; fieldNumber: Biocor 06 kvalita; eventRemarks: M. Svitok col.; **Record Level:** type: PhysicalObject; institutionCode: TUZVO; basisOfRecord: PreservedSpecimen
- **Type status:** Other material. **Taxon:** genus: Martarega; specificEpithet: gonostyla; **Location:** continent: South America; country: Venezuela; stateProvince: Bolívar; county: Gran Sabana; locality: Canaima National Park; decimalLatitude: 5.28636; decimalLongitude: -61.11033; **Identification:** identifiedBy: Juliana F. Barbosa; **Event:** year: 2012; month: 12; day: 4; fieldNumber: Biocor 16 kvalita; eventRemarks: M. Svitok col.; **Record Level:** type: PhysicalObject; institutionCode: TUZVO; basisOfRecord: PreservedSpecimen

#### Distribution

Venezuela!, Suriname, Brazil, Bolivia.

**Distribution in Venezuela**: Bolívar!.

## Discussion

Currently, the knowledge about Gerromorpha and Nepomorpha from Venezuela is based almost entirely on original descriptions of species or records scattered throughout the literature. A checklist of species from the country does not exist, which can be explained by the lack of local taxonomists and by the difficult access to some areas of the country. Based on material from Canaima National Park and its vicinity, it was possible to fill gaps in the geographic distributions of *Brachymetra lata, Cylindrostethus palmaris*, *Limnogonus hyalinus*, *Rhagovelia elegans*, *R. tenuipes*, and *Ambrysus stali*. Expansions to the distribution limits of *R. evidis* (North), *Tenagobia peruana* (North and East), *Limnocoris burmeisteri* (Northwest), *L. fittkaui fittkaui* (North), *Placomerus micans* (North and West), and *Martarega gonostyla* (Northwest) were also detected. In turn, *Rhagovelia yanomamo* and *Ambrysus montandoni* had already been recorded from localities situated in close proximity to the park and were represented in our samples.

Peru has a similar state of knowledge about aquatic and semiaquatic Heteroptera, also due to the lack of specialists and existence of unexplored regions. The few specimens reported here represent North and West expansions to the distribution of *Euvelia lata* and the confimation of the occurrence of *Microvelia pulchella*, the latter previously recorded without exact localities by Drake and Plaumann (1955). On the other hand, some regions of Brazil, especially the states of Rio de Janeiro and Minas Gerais, are very well studied (Moreira et al. 2011). The records presented here concern the local distributions of *Halobatopsis platensis*, *Rhagovelia triangula*, *Limnocoris brasiliensis*, *L. volxemi*, and *Martarega bentoi*.

## Supplementary Material

XML Treatment for Brachymetra lata

XML Treatment for Cylindrostethus palmaris

XML Treatment for Halobatopsis platensis

XML Treatment for Limnogonus hyalinus

XML Treatment for Euvelia lata

XML Treatment for Microvelia pulchella

XML Treatment for Rhagovelia elegans

XML Treatment for Rhagovelia evidis

XML Treatment for Rhagovelia tenuipes

XML Treatment for Rhagovelia triangula

XML Treatment for Rhagovelia yanomamo

XML Treatment for Tenagobia (Romanogobia) peruana

XML Treatment for Ambrysus montandoni

XML Treatment for Ambrysus stali

XML Treatment for Limnocoris brasiliensis

XML Treatment for Limnocoris burmeisteri

XML Treatment for Limnocoris fittkaui fittkaui

XML Treatment for Limnocoris volxemi

XML Treatment for Placomerus micans

XML Treatment for Martarega bentoi

XML Treatment for Martarega gonostyla

## Figures and Tables

**Table 1.** Collecting localities of Gerromorpha and Nepomorpha in South America.

**Country**	**State / Province**	**Municipality / County**	**Locality**	**Latitude**	**Longitude**
Venezuela	Bolívar	Gran Sabana	Canaima National Park, Biocor 01	5.67315	-61.40467
Venezuela	Bolívar	Gran Sabana	Canaima National Park, Biocor 02	5.65003	-61.39197
Venezuela	Bolívar	Gran Sabana	Canaima National Park, Biocor 03	4.91728	-61.09222
Venezuela	Bolívar	Gran Sabana	Canaima National Park, Biocor 04	4.89658	-61.09136
Venezuela	Bolívar	Gran Sabana	Canaima National Park, Biocor 06	4.86188	-61.10061
Venezuela	Bolívar	Gran Sabana	Canaima National Park, Biocor 08	4.94219	-61.08764
Venezuela	Bolívar	Gran Sabana	Canaima National Park, Biocor 10	5.03508	-60.97569
Venezuela	Bolívar	Gran Sabana	Canaima National Park, Biocor 11	5.03656	-61.07594
Venezuela	Bolívar	Gran Sabana	near Canaima National Park, Biocor 12	4.70389	-61.29169
Venezuela	Bolívar	Gran Sabana	Canaima National Park, Biocor 13	4.70000	-61.33269
Venezuela	Bolívar	Gran Sabana	Canaima National Park, Biocor 14	4.63033	-61.32733
Venezuela	Bolívar	Gran Sabana	Canaima National Park, Biocor 15	5.15958	-61.10431
Venezuela	Bolívar	Gran Sabana	Canaima National Park, Biocor 16	5.28636	-61.11033
Venezuela	Bolívar	Gran Sabana	Canaima National Park, Biocor 17	5.21005	-61.09400
Venezuela	Bolívar	Gran Sabana	Canaima National Park, Biocor 18	5.57708	-61.31242
Venezuela	Bolívar	Gran Sabana	Canaima National Park, left side tributary below Salto del Danto	5.96433	-61.38264
Brazil	Bahia	Lençóis	Rio Lençóis, BR 8/2011	-12.56014	-41.40442
Brazil	Bahia	Lençóis	Rio Lençóis, BR 11/2011	-12.55894	-41.40489
Brazil	Minas Gerais	Alto Caparaó	Vale Verde, BR 7/2011	-20.42000	-41.84486
Brazil	Minas Gerais	Alto Caparaó	Rio Caparaó	-20.43300	-41.86672
Peru	Loreto	Iquitos (Maynas)	Río Momón, Lores	-3.51123	-73.40319
Peru	Arequipa	La Unión	Cotahuasi Canyon, Laguna Chaquicocha	-15.20453	-72.89195
